# Prognostic significance of blood pressure parameters after mechanical thrombectomy according to collateral status

**DOI:** 10.1186/s12883-023-03160-3

**Published:** 2023-03-28

**Authors:** Huaishun Wang, Huihui Liu, Qianmei Jiang, Shoujiang You, Zhiliang Guo, Jie Hou, Guodong Xiao

**Affiliations:** Department of Neurology and Suzhou Clinical Research Center of Neurological Disease, the Second Affiliated Hospital of Soochow University, No. 1055 Sanxiang Road, Suzhou, 215004 China

**Keywords:** Ischemic stroke, Blood pressure, Mechanical thrombectomy, Collateral status, Outcome

## Abstract

**Background:**

Mechanical thrombectomy (MT) has been proven as an effective and safe therapy for patients with acute ischemic stroke from large vessel occlusion. However, there is still a controversial topic about post-procedural management including blood pressure (BP).

**Methods:**

A total of 294 patients who received MT in Second Affiliated Hospital of Soochow University from April 2017 to September 2021 were included consecutively. The association of blood pressure parameters (BPV and hypotension time) with poor functional outcome was evaluated using logistic regression models. Meanwhile, the effects of BP parameters on mortality was analyzed using cox proportional hazards regression models. Furthermore, the corresponding multiplicative term was added to the above models to study the interaction between BP parameters and CS.

**Results:**

Two hundred ninety four patients were included finally. The mean age was 65.5 years. At the 3-month follow-up, 187(61.5%) had poor functional outcome and 70(23.0%) died. Regardless of the CS, BP CV is positively associated with poor outcome. Hypotension time was negatively associated with poor outcome. We conducted a subgroup analysis according to CS. BPV was significantly associated with mortality at 3-month and displayed a trend toward poor outcome for patients with poor CS only. The interaction between SBP CV and CS with respect to mortality after adjusting for confounding factors was statistically significant (*P* for interaction = 0.025) and the interaction between MAP CV and CS with respect to mortality after multivariate adjustment was also statistically significant (*P* for interaction = 0.005).

**Conclusion:**

In MT-treated stroke patients, higher BPV in the first 72 h is significantly associated with poor functional outcome and mortality at 3-month regardless of CS. This association was also found for hypotension time. Further analysis showed CS modified the association between BPV and clinical prognosis. BPV displayed a trend toward poor outcome for patients with poor CS.

**Supplementary Information:**

The online version contains supplementary material available at 10.1186/s12883-023-03160-3.

## Introduction

In recent years, the incidence of acute ischemic stroke with large vessel occlusion (LVO) has increased significantly [1]. Mechanical thrombectomy (MT) has gradually changed the post-stroke treatment model [2–6]. Blood pressure (BP) play a key role in patients received MT. American Heart Association/American Stroke Association recommends that blood pressure below 180/105 mmHg. Higher BP level and BP variability (BPV) after acute ischemic stroke is associated with poor prognosis [7, 8]. Impaired autonomic regulation makes patients more susceptible to BP fluctuation after ischemic stroke, which aggravates or leads to reperfusion injury in the infarct area [9, 10].

In 2018, DAWN and DEFFUSE 3 extended the time windows of MT from 6 to 24 h by estimating the volume of ischemic penumbral tissue [11, 12]. This reflects the importance of collateral status (CS). CS is the key to maintaining the perfusion of the ischemic penumbra and preventing further expansion of core infarct volume [13]. Elevated BP may help to maintain collateral flow and reduce the final infarct volume. However, hypertension may also increase the risk of cerebral edema and hemorrhagic transformation [14]. According to the pathophysiological mechanism, the impact of BP on the prognosis may be related to CS, which affects the size of ischemic penumbra tissue and ultimately leading poor outcome [15, 16].

In summary, there is still a controversy regarding BP indicators and prognosis after MT in patients with acute ischemic stroke. Current studies mainly focus on the measurement of systolic blood pressure (SBP) indicators. However, SBP cannot reflect overall situation. Our study aims to exploring the association of postprocedural BP indicators, and further analyze the impact of CS. All data generated or analysed during this study are included in this published article and its Supplementary information files.

## Methods

### Study population and setting

We retrospectively analyzed a consecutive series of stroke patients with LVO and they were treated with MT. In the end, a total of 294 patients were included. The exclusion criteria are as follows: (1) known prestroke mRS > 1, (2) intracranial hemorrhage or arteriovenous malformations were confirmed by CT, (3) terminal medical diagnoses such as a stage IV cancer, (4) the ASPECT score obtained by preoperative CT is less than 6 points or (5) 3-month follow-up data is missing. All methods were carried out in accordance with relevant guidelines and regulations.

### Baseline demographic and clinical information

The baseline information included demographic information, medical history, clinical features, time from onset to vessel recanalization, and imaging features. Medical history included history of hypertension, diabetes mellitus, prior stroke, atrial fibrillation, current smoking, drinking. Clinical features included blood pressure and heart rate profile on admission, baseline National Institutes of Health Stroke Scale (NIHSS), the Alberta Stroke Program Early CT Score (ASPECTS). Imaging features included the site of the occluded brain artery (ICA with or without MCA/ACA) isolated MCA or ACA, and vertebrobasilar or other location), the CS before procedure and reperfusion status after procedure. Collateral status was assessed blind using the American Society of Interventional and Therapeutic Neuroradiology/Society of Interventional Radiology (ASITN/SIR) grading by two neurologists and corrected by a third neurologist if the results were inconsistent [17]. ASITN 0–1 are defined as poor CS and 2–4 as good CS [18]. The etiologic subtypes of stroke were defined according to the Trial of ORG 10,172 in acute stroke treatment (TOAST). The cerebral tissue reperfusion was evaluated by modified Thrombolysis in Cerebral Infarction (mTICI) scale and classified as no perfusion (grade 0), minimal perfusion (Grade 1), partial perfusion (Grade 2: a < 2⁄3 of the entire vascular territory; b complete filling but slowly) and complete perfusion [19]. The state of grade 2b/3 was generally regarded as successful reperfusion.

BP values of patients were routinely monitored by electrocardiograph monitor after MT procedure during hospitalization in stroke units or neuro critical care units and entered into the electronic medical records. We acquired the initial 72 h of hourly heart rate information after MT therapy and calculated BPV using 2 statistical methodologies, i.e. standard deviation (SD) and coefficient of variation (CV).

$$\begin{array}{cc}\mathrm{SD}=\sqrt{\left(\frac1{n-1}\right)\sum\nolimits_{(i=1)}^{(n)}{({BP}_i-{BP}_{mean})}^2}&\mathrm{CV}=\left(\frac{\mathrm{SD}}{{\mathrm{BP}}_{\mathrm{mean}}}\right)\ast100\end{array}.$$

### Outcome assessment

Follow-up was conducted by the trained neurologists who were blinded to the baseline information of patients by telephone or face-to-face visit. The primary outcome events were as follows: (1) poor functional outcome at 3-month (mRS ≥ 3 points), (2) all caused mortality at 3-month (mRS = 6 points).

### Statistical analysis

Continuous variables were appropriately expressed as means with standard deviation (SD) or medians with interquartile range (IQR) and were analyzed by the Student *t* test or Mann–Whitney *U* test according to their normality of distribution. Categorical variables were presented as proportions and analyzed by the χ2 or Fisher exact tests.

For part 1, BP indicators are analyzed as a continuous variable. We used logistic regression models to assess the association of functional outcome event with BP indicators. Cox regression models were used to assess the association of mortality with BP variables.

For part 2, the effect of CS was tested using interaction model with BPV. Then we performed a subgroup analysis for the association of BPV with outcomes according to CS. A two-tailed *P* < 0.05 was considered as significant in this study. All statistics were conducted with SAS 9.4 software (SAS Institute Inc., Cary, NC). Figures were drawn by R software (R Development Core Team 2014, www.r-project.org).

## Results

A total of 294 LVO patients were finally included with the mean age was 65.5 ± 13.7 years old. 107 patients had a good functional outcome and 187 patients had a poor functional outcome (Table 1). The median baseline NIHSS score was 16 (13–20). Patients who had a poor functional outcome were more likely to be elderly, male, higher baseline NIHSS score, higher baseline ASPECTS score, higher admission SBP, higher admission blood glucose level, less vessel recanalization and poor CS. Comparison of data of surviving and deceased patients is shown in Table 2.

**Table 1:** Baseline Demographics of Entire Cohort, and Compared Between MT-Treated Patients With good (mRS, 0–2) Versus poor outcome (mRS, 3–6)

Variable	*N* = 294	good (mRS 0–2) *N* = 107	poor (mRS 3–6) *N* = 187	*P*
Age, mean ± SD	65.5 ± 13.7	60.1 ± 15.2	68.6 ± 11.8	** < 0.001**
Male sex, n, %	167 (56.8)	74 (69.2)	93 (49.7)	**0.001**
Baseline NIHSS (median, IQR)	16 (13–20)	13 (11–17)	17 (14–21)	** < 0.001**
Baseline ASPECTS (median, IQR)	7 (7–8)	7 (7–8)	7 (6–7)	** < 0.001**
SBP at admission, mmHg, mean ± SD	144.1 ± 21.9	140.7 ± 19.2	146.1 ± 23.2	**0.049**
DBP at admission, mmHg, mean ± SD	83.9 ± 15.9	84.2 ± 14.6	83.7 ± 16.6	0.121
Heart rate at admission, beat per minute, mean ± SD	83.1 ± 20.2	81.1 ± 18.4	84.2 ± 21.2	0.055
glucose level, mg/dL, mean ± SD	8.04 ± 2.77	7.3 ± 2.3	8.5 ± 2.9	**0.016**
History of hypertension, n, %	141 (68.5)	67 (62.6)	133 (71.1)	0.132
History of diabetes mellitus, n, %	53 (18.0)	15 (14.0)	38 (20.3)	0.176
History of atrial fibrillation, n, %	131 (44.6)	41 (38.3)	90 (48.1)	0.103
History of coronary heart disease, n, %	38 (12.9)	12 (11.2)	26 (13.9)	0.508
History of prior stroke, n, %	37 (12.6)	10 (9.3)	27 (14.4)	0.205
Previous smoking habit, n, %	103 (35.2)	46 (43.0)	57 (30.6)	0.167
Previous drinking habit, n, %	68 (23.2)	30 (28.0)	38 (20.4)	0.138
Suspected stroke cause				0.648
Large-artery atherosclerosis, n, %	124 (42.0)	42 (39.3)	82 (43.6)	
Cardioembolic, n, %	151 (51.2)	52 (48.6)	99 (52.7)	
Others, n, %	20 (6.8)	13 (12.1)	7 (3.7)	
Site of occlusion				0.375
ICA with or without MCA, n, %	38 (12.9)	10 (9.3)	28 (14.9)	
MCA, n, %	222 (75.3)	83 (77.6)	139 (73.9)	
VA or BA, n, %	35 (11.9)	14 (12.3)	13 (9.8)	
tPA administered, n, %	94 (32.0)	38 (35.5)	56 (29.9)	0.325
Minutes from stroke door to puncture, mean ± SD	79 (45–133)	81 (42–152)	79 (48–129)	0.946
Minutes from stroke puncture to recanalization, mean ± SD	60 (40–85)	57 (40–81)	60 (38–87)	0.740
Minutes from stroke onset to recanalization, mean ± SD	350 (272–446)	358 (273–446)	350 (270–447)	0.852
Recanalization (TICI 2b–3), n, %	252 (85.7)	101 (95.3)	151 (80.3)	** < 0.001**
Good CS (ASITN/SIR 2–4), n, %	185 (63.1)	95 (90.5)	90 (47.9)	** < 0.001**

**Table 2:** Baseline Demographics of Entire Cohort, and Compared Between MT-Treated Patients With alive (mRS, 0–5) Versus dead outcome (mRS, 6)

Variable	*N* = 294	alive (mRS 0–5) *N* = 224	dead (mRS 6) *N* = 70	*P*
Age, mean ± SD	65.5 ± 13.7	63.6 ± 14.2	71.4 ± 10.3	**0.001**
Male sex, n, %	167 (56.8)	132 (58.9)	35 (50.0)	0.188
Baseline NIHSS (median, IQR)	16 (13–20)	15 (12–18)	20 (16–25)	** < 0.001**
Baseline ASPECTS (median, IQR)	7 (7–8)	7 (7–8)	7 (6–7)	** < 0.001**
SBP at admission, mmHg, mean ± SD	144.1 ± 21.9	142.5 ± 19.5	149.6 ± 27.9	** < 0.001**
DBP at admission, mmHg, mean ± SD	83.9 ± 15.9	83.4 ± 14.4	87.6 ± 19.2	** < 0.001**
Heart rate at admission, beat per minute, mean ± SD	83.1 ± 20.2	82.5 ± 19.7	85.1 ± 21.9	0.292
glucose level, mg/dL, mean ± SD	8.04 ± 2.77	7.6 ± 2.5	9.4 ± 3.3	**0.019**
History of hypertension, n, %	141 (68.5)	141 (62.9)	59 (84.3)	**0.001**
History of diabetes mellitus, n, %	53 (18.0)	34 (15.2)	19 (27.1)	0.072
History of atrial fibrillation, n, %	131 (44.6)	100 (44.6)	31 (44.3)	0.958
History of coronary heart disease, n, %	38 (12.9)	26 (11.6)	12 (17.1)	0.228
History of prior stroke, n, %	37 (12.6)	29 (12.9)	8 (11.4)	0.738
Previous smoking habit, n, %	103 (35.2)	80 (35.9)	23 (32.9)	0.645
Previous drinking habit, n, %	68 (23.2)	52 (23.3)	16 (22.9)	0.936
Suspected stroke cause				0.559
Large-artery atherosclerosis, n, %	124 (42.0)	92 (40.9)	32 (45.7)	
Cardioembolic, n, %	151 (51.2)	116 (51.6)	35 (50.0)	
Others, n, %	20 (6.8)	17 (7.6)	3 (4.3)	
Site of occlusion				0.078
ICA with or without MCA, n, %	38 (12.9)	25 (11.1)	13 (18.6)	
MCA, n, %	222 (75.3)	179 (79.6)	43 (61.4)	
VA or BA, n, %	35 (11.9)	13 (8.5)	9 (17.0)	
tPA administered, n, %	94 (32.0)	75 (33.5)	19 (27.1)	0.321
Minutes from stroke door to puncture, mean ± SD	79 (45–133)	90 (47–142.5)	60 (37–105)	**0.021**
Minutes from stroke puncture to recanalization, mean ± SD	60 (40–85)	60 (40–85)	55 (36–87)	0.686
Minutes from stroke onset to recanalization, mean ± SD	350 (272–446)	356 (275–460)	338 (265–434)	0.259
Recanalization (TICI 2b–3), n, %	252 (85.7)	200 (89.3)	52 (74.3)	**0.002**
Good CS (ASITN/SIR 2–4), n, %	185 (63.1)	165 (74.0)	20 (28.6)	** < 0.001**

### Association of BP measures with outcomes

As shown in Table 3, the BP index is used as a continuous variable. The risk of poor functional outcome increases by 40% when mean SBP increased by 10 mmHg(95%CI: 1.18–1.67, *P* < 0.001). Similar relationships are also found in PP, and MAP. SBP SD and SBP CV are significantly associated with poor functional outcome. Whether for SBP, DBP or MAP, the longer hypotension time, the lower risk of poor functional outcome. After adjusting for age, gender, baseline NIHSS, baseline ASPECTS, admission SBP, glucose and degree of recanalization, the risk of poor functional outcome increased by 0.91 (*P* = 0.037). The same relationship was found in SBP SD with an odds ratio of 1.20–3.24. We also found mean MAP and time with MAP < 90 mmHg are both associated with poor functional outcome. In Table 4, we explored the association of BP indicators with 3-month all caused mortality. In the cox regression model, the mean SBP, SBP SD and SBP CV all predicted mortality. These associations were still statistically significant in Model 1. In the multivariate model that adjusted for age, baseline NIHSS, baseline ASPECTS, admission SBP, glucose level, history of hypertension, tPA use, and vessel recanalization degree, SBP SD and SBP CV remained significantly associated with mortality at 3-month. In the cox regression model, time with SBP < 140 mmHg consistently remained as a predictor of mortality.

**Table 3:** Logistic regression analysis of BP indicators (continuous variable) and poor outcome (mRS ≥ 3)

Variable	Unadjusted		Model 1		Model 2	
	**OR(95% CI)**	***P***	**OR(95% CI)**	***P***	**OR(95% CI)**	***P***
SBP						
Mean SBP (per 10 mmHg)	1.40 (1.18–1.67)	** < 0.001**	1.32(1.11–1.57)	**0.002**	1.27 (1.01–1.61)	**0.045**
SBP SD (per 5 unit)	2.58(1.74–3.84)	** < 0.001**	2.13(1.45–3.13)	**0.001**	1.98(1.20–3.24)	**0.007**
SBP CV (per 5 unit)	2.62(1.60–4.30)	** < 0.001**	2.15(1.34–3.45)	**0.002**	1.91(1.04–3.50)	**0.037**
DBP						
Mean DBP (per 10 mmHg)	1.26(0.97–1.63)	0.081	1.53(1.15–2.03)	**0.004**	1.59(1.10–2.29)	**0.013**
DBP SD (per 5 unit)	1.16(0.93–1.46)	0.192	1.13(0.96–1.34)	0.151	1.08(0.92–1.26)	0.371
DBP CV (per 5 unit)	1.12(0.96–1.32)	0.155	1.09(0.96–1.24)	0.200	1.05(0.91–1.21)	0.509
PP						
Mean PP (per 10 mmHg)	1.31(1.10–1.56)	**0.003**	1.15(0.96–1.40)	0.138	0.99(0.77–1.28)	0.953
PP SD (per 5 unit)	1.23(0.99–1.53)	0.061	1.16(0.99–1.35)	0.059	1.12(0.96–1.29)	0.150
PP CV (per 5 unit)	1.02(0.98–1.07)	0.324	1.03(0.99–1.07)	0.159	1.03(0.98–1.07)	0.244
MAP						
Mean MAP (per 10 mmHg)	1.21(1.01–1.45)	**0.043**	1.24(1.03–1.49)	**0.027**	1.17(0.92–1.49)	0.199
MAP SD (per 5 unit)	1.20(1.02–1.42)	**0.033**	1.19(1.02–1.39)	**0.025**	1.18(0.98–1.42)	0.087
MAP CV (per 5 unit)	1.06(0.98–1.15)	0.146	1.06(0.98–1.15)	0.148	1.07(0.97–1.18)	0.202
Hypotension time						
Percentage of SBP < 140 mm Hg (per 10 percentage)	0.85(0.77–0.93)	** < 0.001**	0.87(0.79–0.96)	**0.004**	0.91(0.80–1.04)	0.154
Percentage of DBP < 70 mm Hg (per 10 percentage)	0.93(0.85–1.02)	0.106	0.88(0.80–0.97)	**0.008**	0.85(0.75–0.96)	**0.011**
Percentage of MAP < 90 mm Hg (per 10 percentage)	0.88(0.81–0.95)	**0.002**	0.86(0.79–0.95)	**0.002**	0.85(0.75–0.96)	**0.009**

Model 1: Adjust age and gender

Model 2: Adjust age, gender, baseline NIHSS, baseline ASPECTS, SBP at admission, glucose level at admission and recanalization degree

**Table 4:** Cox regression analysis of BP indicators (continuous variable) and mortality (mRS 6)

Variable	Unadjusted		Model 1		Model 2	
	**HR(95% CI)**	***P***	**HR(95% CI)**	***P***	**HR(95% CI)**	***P***
SBP						
Mean SBP (per 10 mmHg)	1.40(1.19–1.65)	** < 0.001**	1.35(1.13–1.60)	** < 0.001**	1.13(0.91–1.41)	0.275
SBP SD (per 5 unit)	1.08(1.04–1.11)	** < 0.001**	1.17(1.04–1.10)	** < 0.001**	1.06(0.99–1.12)	0.065
SBP CV (per 5 unit)	1.11(1.07–1.16)	** < 0.001**	1.10(1.05–1.15)	** < 0.001**	1.09(1.01–1.17)	**0.020**
DBP						
Mean DBP (per 10 mmHg)	0.97(0.75–1.25)	0.824	1.09(0.84–1.43)	0.524	0.93(0.66–1.29)	0.645
DBP SD (per 5 unit)	1.06(1.01–1.11)	**0.022**	1.06(1.01–1.12)	**0.013**	1.11(0.99–1.24)	0.089
DBP CV (per 5 unit)	1.06(1.02–1.11)	**0.003**	1.06(1.02–1.11)	**0.004**	1.09(1.01–1.17)	**0.027**
PP						
Mean PP (per 10 mmHg)	1.34(1.14–1.59)	** < 0.001**	1.21(1.01–1.45)	**0.040**	1.00(0.80–1.25)	0.996
PP SD (per 5 unit)	1.08(1.03–1.14)	**0.003**	1.11(1.05–1.17)	** < 0.001**	1.15(1.04–1.28)	**0.008**
PP CV (per 5 unit)	1.03(1.01–1.05)	**0.014**	1.05(1.02–1.07)	** < 0.001**	1.06(1.03–1.09)	** < 0.001**
MAP						
Mean MAP (per 10 mmHg)	0.95(0.78–1.15)	0.593	0.93(0.76–1.15)	0.525	0.96(0.91–1.56)	0.145
MAP SD (per 5 unit)	1.19(1.11–1.28)	** < 0.001**	1.26(1.16–1.37)	** < 0.001**	1.34(1.19–1.50)	** < 0.001**
MAP CV (per 5 unit)	1.09(1.06–1.13)	** < 0.001**	1.10(1.11–1.41)	** < 0.001**	1.12(1.08–1.17)	** < 0.001**
Hypotension time						
Percentage of SBP < 140 mm Hg (per 10 percentage)	0.85(0.79–0.91)	** < 0.001**	0.86(0.80–0.93)	** < 0.001**	0.90(0.82–0.99)	**0.048**
Percentage of DBP < 70 mm Hg (per 10 percentage)	1.03(0.94–1.12)	0.565	1.00(0.91–1.09)	0.937	1.04(0.94–1.16)	0.460
Percentage of MAP < 90 mm Hg (per 10 percentage)	0.93(0.86–1.01)	0.090	0.93(0.85–1.01)	0.092	0.99(0.88–1.11)	0.817

Model 1: Adjust age and gender

Model 2: Adjust age, baseline NIHSS, baseline ASPECTS, SBP at admission, glucose level at admission, history of hypertension, tPA administered, and recanalization degree

**Fig. 1:**
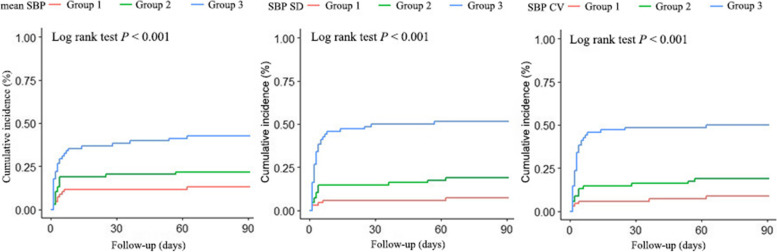
The association of BP values and mortality

Taking SBP as an example, all patients were equally divided into three groups according to the level of SBP index, that is, SBP was used as a categorical variable to explore the association. In the logistic model, mean SBP, SBP SD and SBP CV were all significantly associated poor functional outcome (all *P* for trend < 0.001) in Table 5. After adjusting for confounding factors, we could observe elevated SBP SD and SBP CV increase the risk of poor outcome. In the cox regression model, SBP SD and SBP CV consistently remained associated with 3-month mortality as shown in Table 6. Kaplan–Meier survival curves were performed to estimate the association of mean SBP, SBP SD, SBP CV and mortality in Fig. 1.

**Table 5:** Logistic regression analysis of BP indicators (categorical variables) and poor outcome (mRS ≥ 3)

Variable	*N* = 206	Unadjusted		Model 1		Model 2	
		**OR(95% CI)**	***P* for trend**	**OR(95% CI)**	***P* for trend**	**OR(95% CI)**	***P* for trend**
Mean SBP			** < 0.001**		**0.003**		0.065
Group 1	69	1.00		1.00		1.00	
Group 2	69	2.99(1.65–5.39)		2.58(1.38–4.80)		2.47(1.13–5.40)	
Group 3	68	3.26(1.79–5.94)		2.55(1.35–4.80)		2.21(0.92–5.32)	
SBP SD			** < 0.001**		** < 0.001**		**0.021**
Group 1	69	1.00		1.00		1.00	
Group 2	69	2.15(1.22–3.82)		1.77(0.97–3.23)		1.48(0.70–3.14)	
Group 3	68	4.90(2.58–9.28)		3.64(1.85–7.16)		2.78(1.17–6.59)	
SBP CV			** < 0.001**		** < 0.001**		**0.031**
Group 1	69	1.00		1.00		1.00	
Group 2	69	2.37(1.33–4.22)		2.00(1.09–3.67)		1.95 (0.94–4.08)	
Group 3	68	3.69(2.00–6.80)		2.93(1.54–5.55)		2.36(1.06–5.27)	

Mean SBP Group 1: Mean SBP < 122.3 mmHg Group 2: 122.3 mmHg ≤ Mean SBP < 137.6 mmHg Group 3: Mean SBP ≥ 137.6 mmHg

SBP SD Group 1: SBP SD < 10.5 Group 2: 10.5 ≤ SBP SD < 13.3 Group 3: SBP SD ≥ 13.3

SBP CV Group 1: SBP CV < 8.2 Group 2: 8.2 ≤ SBP CV < 10.4 Group 3: SBP CV ≥ 10.4

Model 1: Adjust age and gender

Model 2: Adjust age, gender, baseline NIHSS, baseline ASPECTS, SBP at admission, glucose level at admission and recanalization degree

**Table 6:** Cox regression analysis of BP indicators (categorical variables) and mortality (mRS 6)

Variable	*N* = 206	Unadjusted		Model 1		Model 2	
		**HR(95% CI)**	***P* for trend**	**HR(95% CI)**	***P* for trend**	**HR(95% CI)**	***P* for trend**
Mean SBP			** < 0.001**		** < 0.001**		0.132
Group 1	69	1.00		1.00		1.00	
Group 2	69	2.56(1.22–5.35)		2.23(1.06–4.67)		1.46(0.64–3.34)	
Group 3	68	4.13(2.05–8.33)		3.29(1.62–6.67)		1.89(0.81–4.41)	
SBP SD			** < 0.001**		** < 0.001**		** < 0.001**
Group 1	69	1.00		1.00		1.00	
Group 2	69	3.81(1.41–10.26)		3.32(1.23–9.01)		2.17(0.76–6.22)	
Group 3	68	12.01(4.77–30.23)		9.80(3.83–25.09)		5.73(2.14–15.34)	
SBP CV			** < 0.001**		** < 0.001**		** < 0.001**
Group 1	69	1.00		1.00		1.00	
Group 2	69	2.99(1.27–7.08)		2.58(1.08–6.12)		2.14(0.77–5.94)	
Group 3	68	7.56(3.40–16.83)		6.42(2.87–14.35)		4.91(1.87–12.85)	

Mean SBP Group 1: Mean SBP < 122.3 mmHg Group 2: 122.3 mmHg ≤ Mean SBP < 137.6 mmHg Group 3: Mean SBP ≥ 137.6 mmHg

SBP SD Group 1: SBP SD < 10.5 Group 2: 10.5 ≤ SBP SD < 13.3 Group 3: SBP SD ≥ 13.3

SBP CV Group 1: SBP CV < 8.2 Group 2: 8.2 ≤ SBP CV < 10.4 Group 3: SBP CV ≥ 10.4

Model 1: Adjust age and gender

Model 2: Adjust age, baseline NIHSS, baseline ASPECTS, SBP at admission, glucose level at admission, history of hypertension, tPA administered, and recanalization degree

### Association of BP measures with outcomes according to CS

As shown in Table 7, there were 185 patients with good CS (ASITN 2–4 points) and 109 patients with poor CS (ASITN 0–1 points). We observed time with DBP < 70 mmHg and MAP < 90 mmHg had a significant negative association with poor functional outcome in patients with poor CS. After adjusting for multiple factors, for per 10 percent increase in the time of DBP < 70 mmHg, the odds ratio were 0.85 (95%*CI*: 0.73–0.99, *P* = 0.034). There is no evidence that CS and duration of hypotension have an interactive effect on poor functional outcome. In patients with poor CS, there is a significant association of SBP CV, DBP CV and MAP CV with functional outcome. Notably, the association between SBP CV, DBP CV and MAP CV with CS reached a significant level statistically.

**Table 7:** The impact of BP indicators (BPV and hypotension time) on poor outcome according to Collateral status

**Variable**	**Collateral**	**Unadjusted**	**Adjusted**		
	**Status (CS)**	**OR (95%Cl)**	**OR (95%Cl)**	***P* value**	***P* for interaction**
**BP variability**					
SBP CV					0.025
	Good	1.13(1.00–1.26)	1.07(0.93–1.25)	0.351	
	Poor	1.51(1.08–2.13)	2.20(1.07–4.51)	0.033	
DBP CV					0.023
	Good	1.01(0.98–1.04)	1.01(0.98–1.04)	0.568	
	Poor	1.26(0.98–1.62)	1.73(1.04–2.89)	0.036	
MAP CV					0.005
	Good	0.99(0.97–1.01)	1.00(0.97–1.03)	0.958	
	Poor	1.52(1.10–2.10)	2.30(1.20–4.39)	0.012	
**Hypotension time (per 10 percentage)**					
Percentage of SBP < 140 mm Hg					0.828
	Good	0.89(0.80–0.99)	0.99(0.84–1.17)	0.888	
	Poor	0.92(0.72–1.16)	0.94(0.69–1.28)	0.686	
Percentage of DBP < 70 mm Hg					0.418
	Good	0.92(0.83–1.02)	0.85(0.73–0.99)	0.034	
	Poor	1.02(0.79–1.32)	0.93(0.64–1.36)	0.708	
Percentage of MAP < 90 mm Hg					0.883
	Good	0.91(0.82–1.00)	0.89(0.77–1.02)	0.110	
	Poor	0.92(0.71–1.18)	0.81(0.56–1.18)	0.275	

Adjust age, gender, baseline NIHSS, baseline ASPECTS, SBP at admission, glucose level at admission and recanalization degree

In Table 8, we couldn’t find the interaction between hypotension time and all caused mortality is significant. SBP CV, DBP CV and MAP CV all exhibited a positive association with mortality (*P* = 0.045, 0.019 and < 0.001). And we observed SBP CV and CS have a significant interaction effect on mortality (*P* for interaction < 0.001).

**Table 8:** The impact of BP indicators (BPV and hypotension time) on mortality according to Collateral status

**Variable**	**Collateral**	**Unadjusted**	**Adjusted**		
	**Status (CS)**	**HR (95%Cl)**	**HR (95%Cl)**	***P* value**	***P* for interaction**
**BP variability**					
SBP CV					< 0.001
	Good	1.26(1.15–1.39)	1.40(1.18–1.65)	< 0.001	
	Poor	1.01(1.00–1.02)	1.02(1.00–1.04)	0.045	
DBP CV					0.522
	Good	1.00(0.96–1.04)	1.00(0.93–1.07)	0.982	
	Poor	1.01(1.00–1.02)	1.02(1.00–1.03)	0.019	
MAP CV					0.801
	Good	0.99(0.95–1.04)	1.03(0.98–1.08)	0.155	
	Poor	1.02(1.01–1.02)	1.02(1.01–1.03)	< 0.001	
**Hypotension time**					
Percentage of SBP < 140 mm Hg					0.290
	Good	0.78(0.68–0.89)	0.80(0.64–1.00)	0.050	
	Poor	0.94(0.86–1.04)	0.93(0.81–1.07)	0.314	
Percentage of DBP < 70 mm Hg					0.925
	Good	1.06(0.91–1.23)	1.04(0.84–1.27)	0.742	
	Poor	1.04(0.94–1.16)	1.07(0.94–1.22)	0.418	
Percentage of MAP < 90 mm Hg					0.619
	Good	0.92(0.79–1.07)	0.95(0.76–1.17)	0.610	
	Poor	1.01(0.90–1.12)	1.04(0.89–1.20)	0.636	

Adjust age, baseline NIHSS, baseline ASPECTS, SBP at admission, glucose level at admission, history of hypertension, tPA administered, and recanalization degree

## Discussion

In this study, higher BPV within 72 h after MT is significantly associated with poor functional outcome and mortality at 3-month. At the same time, we also observed that the hypotension time is associated with outcome. Further analysis of the role of CS, we found the interaction between CS and BPV on endpoint event. And elevated BPV increase the risk of poor outcome. There is no evidence to prove the interaction between hypotension and CS reached significant statistically.

At present, the treatment model of acute ischemic stroke is no longer the traditional "time window" of the past, but has turned to the concept of "tissue window" recognized by more and more scholars [20]. The time of onset to reperfusion determines prognosis of patients with LVO. Thrombolysis was the only hyperacute method of ischemic stroke within 4.5 h of onset [21]. With the advent of MT, the treatment of stroke patients had also moved to a new model. After that, MT was first confirmed to be effective in LVO patients with acute anterior circulation within 6 h [22]. The DAWN and DEFUSE 3 have further expanded the time window of MT, confirming that patients with LVO after imaging screening can benefit even if the onset is more than 6 h. The perioperative management is important. However, the postoperative treatment of MT is still relatively controversial currently [23]. Especially, the management of BP has not yet reached a consensus lack of sufficient high-quality studies.

BP is one of the most important factors of tissue perfusion, and its abnormality is directly related to impaired vascular function. Cerebral blood flow is easily susceptible to BP. After acute ischemic stroke, the cerebral blood flow is almost completely stopped, causing the rapid death of neuronal cells within a few minutes. The penumbra around the core infarct area is severely insufficiently perfused and its function is impaired. But it still maintains a certain activity and is extremely susceptible to BP fluctuations. It was reported that about 75 percentage of patients have elevated BP in the acute phase of stroke [24, 25]. This may be due to physical and psychological stress, increased intracranial pressure, painful stimulation, dehydration, ischemia in key parts of the brain, impaired autonomic nerve function (such as abnormal sympathetic nerve, parasympathetic nerve activity, and increased catecholamines), and baroreceptor sensitivity [9, 10, 26–29]. A lot of studies mentioned that BP may be an important factor related to outcome, and changes in BP directly or indirectly affect the functional prognosis of stroke patients. AHA/ASA recommended that it might be reasonable to control postoperative BP below 180/105 mmHg in patients with LVO, which still lacked support of large clinical randomized controlled trials. Such a high BP level may aggravate brain tissue edema. In order to avoid perfusion injury, DAWN set SBP value to be maintained at 140 mmHg after MT while REVASCAT set BP less than 160/90 mmHg [6, 11]. Due to the heterogeneity of patients, optimal BP level shown by various studies are different.

BPV is defined as change in BP over a period of time, which can reflect the patients’ extreme BP fluctuations. BPV is not affected by BP threshold and has important clinical predictive value. A retrospective study by Bennet et al. included a total of 182 patients who underwent MT. They found that higher BPV increased the risk of poor prognosis for patients at 3 months [30]. Mistry et al. explored that BPV is positively associated with poor outcome, and this is more significant in SBP [31]. This is consistent with our result.

A good CS supplies more blood flow to the ischemic penumbra but a poor CS will make ischemic penumbra more transformed into the core infarct area, and irreversibly damage the brain tissue [32, 33]. Previous studies showed that a good CS reduce the core infarct volume, slow the growth rate of infarction and improve prognosis [34, 35]. Stroke patients with LVO were difficult to benefit from MT. The maintenance of brain tissue activity in the ischemic penumbra depends on CS. At present, there is no relevant high-quality research on whether the association between BP and prognosis changes with CS after MT. We observed higher BP CV had a poor functional outcome, although this did not reach significant statistically. However, we found that BP CV is associated with 3-month mortality significantly. Due to severe cerebral ischemia in patients with a poor CS, systemic BP is required to maintain cerebral blood perfusion. Thus, a decrease or increase of BP may lead to expansion of infarction, bleeding transformation and damage to other organs. In patients with a good CS, autonomic nerve function is not so obviously damaged. The cerebral arterioles and small vessels are still active, which can adjust BP through their own dilation and contraction, so fluctuation of BP is not easy to cause brain tissue damage. This mean that patients with poor CS are more susceptible to blood pressure fluctuations. We can find that MAP CV and CS have an interactive effect on the poor functional outcome, and SBP CV and CS have an interactive effect on mortality. As far as we know, this may be the first time this statistical difference has been confirmed in patients receiving MT, that is CS can change the impact of BPV on outcome. Patients with poor CS usually show more severe neurological deficits [36]. Because infarct volume progresses rapidly, the core infarct volume and peripheral vascular resistance of patients with poor CS is larger than patients with good CS. Thus, increase in BPV may frequently induce hemorrhagic transformation in patients with poor CS. In addition, BP fluctuation can also cause embolism in the vascular system and the ability of patients with poor CS to remove emboli may be lower than that of patients with good CS [37]. The first assessment of CS is before MT. Transfer time may be delayed because of drip-and-shift model of some patients, which will affect the assessment of CS. Therefore, a rapid assessment is necessary, which is helpful for us to perform different BP management in two groups of patients with poor CS and good CS.

Some limitations also exist in our research. First, this is a single-center, retrospective and small sample size study. There are uncertainties in its promotion to clinical practice. Multi-center, large-sample randomized controlled trials are still needed to verify our research results. Second, we cannot avoid selection bias. Some patients lose baseline data, BP data, etc., which makes it impossible for us to include all patients in this study. Third, we lack data related to intraoperative BP. We admit that intraoperative BPV also has a certain impact on the prognosis of patients. After MT, we did not collect information about antihypertensive drugs. Different antihypertensive drugs can also affect BP indicators.

## Conclusion

In summary, this study found that the increased BPV 72 h after MT increased the risk of poor functional outcome and mortality at 3-month. Hypotension time was negatively associated with outcome. We further found that there is an interaction between BPV and CS. Higher BPV was significantly associated with the mortality and exhibited a trend towards functional outcome in patients with poor CS. If interactions are confirmed in subsequent large-sample and multi-center studies, we will be able to perform different BP management after MT according to CS, reducing the risk of poor outcome.

## Supplementary Information

**Additional file 1.**

**Additional file 2.**

## Data Availability

Data can be found in Supplementary File.

## References

[1] Smith W, Lev M, English J (2009). Significance of large vessel intracranial occlusion causing acute ischemic stroke and TIA. Stroke.

[2] Berkhemer O, Fransen P, Beumer D (2015). A randomized trial of intraarterial treatment for acute ischemic stroke. N Engl J Med.

[3] Jovin T, Chamorro A, Cobo E (2015). Thrombectomy within 8 hours after symptom onset in ischemic stroke. N Engl J Med.

[4] Goyal M, Demchuk A, Menon B (2015). Randomized assessment of rapid endovascular treatment of ischemic stroke. N Engl J Med.

[5] Campbell B, Mitchell P, Kleinig T (2015). Endovascular therapy for ischemic stroke with perfusion-imaging selection. N Engl J Med.

[6] Saver J, Goyal M, Bonafe A (2015). Stent-retriever thrombectomy after intravenous t-PA vs. t-PA alone in stroke. N Engl J Med.

[7] de Havenon A, Majersik J, Stoddard G (2018). Increased Blood Pressure Variability Contributes to Worse Outcome After Intracerebral Hemorrhage. Stroke.

[8] de Havenon A, Stoddard G, Saini M (2019). Increased blood pressure variability after acute ischemic stroke increases the risk of death: A secondary analysis of the Virtual International Stroke Trial Archive. JRSM Cardiovasc Dis.

[9] Rose J, Mayer S (2004). Optimizing blood pressure in neurological emergencies. Neurocrit Care.

[10] Bath P, Appleton J, Krishnan K (2018). Blood Pressure in Acute Stroke: To Treat or Not to Treat: That Is Still the Question. Stroke.

[11] Nogueira R, Jadhav A, Haussen D (2018). Thrombectomy 6 to 24 Hours after Stroke with a Mismatch between Deficit and Infarct. N Engl J Med.

[12] Albers G, Marks M, Kemp S (2018). Thrombectomy for Stroke at 6 to 16 Hours with Selection by Perfusion Imaging. N Engl J Med.

[13] Samuels N, van de Graaf R, Dippel D (2019). Letter by Samuels et al Regarding Article, "Decreases in Blood Pressure During Thrombectomy Are Associated With Larger Infarct Volumes and Worse Functional Outcome". Stroke.

[14] Regenhardt R, Das A, Stapleton C (2017). Blood Pressure and Penumbral Sustenance in Stroke from Large Vessel Occlusion. Front Neurol.

[15] Campbell B, Majoie C, Albers G (2019). Penumbral imaging and functional outcome in patients with anterior circulation ischaemic stroke treated with endovascular thrombectomy versus medical therapy: a meta-analysis of individual patient-level data. Lancet Neurol.

[16] Liebeskind D, Tomsick T, Foster L (2014). Collaterals at angiography and outcomes in the Interventional Management of Stroke (IMS) III trial. Stroke.

[17] Liu L, Ding J, Leng X (2018). Guidelines for evaluation and management of cerebral collateral circulation in ischaemic stroke 2017. Stroke Vasc Neurol.

[18] Powers WJ, Rabinstein AA, Ackerson T (2019). Guidelines for the Early Management of Patients With Acute Ischemic Stroke: 2019 Update to the 2018 Guidelines for the Early Management of Acute Ischemic Stroke: A Guideline for Healthcare Professionals From the American Heart Association/American Stroke Association. Stroke.

[19] Zaidat O, Yoo A, Khatri P (2013). Recommendations on angiographic revascularization grading standards for acute ischemic stroke: a consensus statement. Stroke.

[20] Damani R (2018). A brief history of acute stroke care. Aging.

[21] Emberson J, Lees K, Lyden P (2014). Effect of treatment delay, age, and stroke severity on the effects of intravenous thrombolysis with alteplase for acute ischaemic stroke: a meta-analysis of individual patient data from randomised trials. Lancet (London, England).

[22] Goyal M, Menon B, van Zwam W (2016). Endovascular thrombectomy after large-vessel ischaemic stroke: a meta-analysis of individual patient data from five randomised trials. Lancet (London, England).

[23] Leslie-Mazwi T, Chen M, Yi J (2017). Post-thrombectomy management of the ELVO patient: Guidelines from the Society of NeuroInterventional Surgery. J Neurointerv Surg.

[24] Qureshi A, Ezzeddine M, Nasar A (2007). Prevalence of elevated blood pressure in 563,704 adult patients with stroke presenting to the ED in the United States. Am J Emerg Med.

[25] Qureshi A (2008). Acute hypertensive response in patients with stroke: pathophysiology and management. Circulation.

[26] Carcel C, Anderson C (2015). Timing of blood pressure lowering in acute ischemic stroke. Curr Atheroscler Rep.

[27] Sargento-Freitas J, Laranjinha I, Galego O (2015). Nocturnal blood pressure dipping in acute ischemic stroke. Acta Neurol Scand.

[28] Müller M, Österreich M, Müller A (2016). Assessment of the Brain's Macro- and Micro-Circulatory Blood Flow Responses to CO2 via Transfer Function Analysis. Front Physiol.

[29] Lang E, Lagopoulos J, Griffith J (2003). Cerebral vasomotor reactivity testing in head injury: the link between pressure and flow. J Neurol Neurosurg Psychiatry.

[30] Bennett A, Wilder M, McNally J (2018). Increased blood pressure variability after endovascular thrombectomy for acute stroke is associated with worse clinical outcome. J Neurointerv Surg.

[31] Mistry E, Mehta T, Mistry A (2020). Blood Pressure Variability and Neurologic Outcome After Endovascular Thrombectomy: A Secondary Analysis of the BEST Study. Stroke.

[32] Man S, Aoki J, Hussain M (2015). Predictors of infarct growth after endovascular therapy for acute ischemic stroke. J Stroke Cerebrovasc Dis.

[33] Puhr-Westerheide D, Tiedt S, Rotkopf L (2019). Clinical and Imaging Parameters Associated With Hyperacute Infarction Growth in Large Vessel Occlusion Stroke. Stroke.

[34] Jung S, Gilgen M, Slotboom J (2013). Factors that determine penumbral tissue loss in acute ischaemic stroke. Brain J Neurol.

[35] Payabvash S, Taleb S, Qureshi A (2017). Cerebral regions preserved by successful endovascular recanalization of acute M1 segment occlusions: a voxel based analysis. Br J Radiol.

[36] Nambiar V, Sohn S, Almekhlafi M (2014). CTA collateral status and response to recanalization in patients with acute ischemic stroke. AJNR Am J Neuroradiol.

[37] Caplan L, Hennerici M (1998). Impaired clearance of emboli (washout) is an important link between hypoperfusion, embolism, and ischemic stroke. Arch Neurol.

