# Supplementary material for: Prognostic significance of blood pressure parameters after mechanical thrombectomy according to collateral status
Source: BMC Neurol. 2023 Mar 28;23:123. doi: 10.1186/s12883-023-03160-3 (PMC10044781; doi:10.1186/s12883-023-03160-3)
Supplement: Supplementary file 1 — Additional file 1. [file 12883_2023_3160_MOESM1_ESM.docx]

**BP values in good and poor outcomes group**

| **Variable** | **good**  **(mRS 0-2)**  **N=107** | **poor**  **(mRS 3-6)**  **N=187** |
| --- | --- | --- |
| Mean SBP | 122.2 (113.9-136.4) | 131.8 (123.0-142.1) |
| SBP SD | 10.8 (9.0-12.8) | 12.7 (10.9-17.0) |
| SBP CV | 8.3 (7.5-10.1) | 9.9 (8.3-12.4) |
| Mean DBP | 72.8 (65.8-78.1) | 75.1 (67.5-80.0) |
| DBP SD | 8.9 (7.9-10.4) | 9.8 (8.4-11.8) |
| DBP CV | 12.2 (10.8-14.4) | 13.0 (11.2-15.7) |
| Mean MAP | 89.3 (82.3-96.9) | 92.4 (87.2-99.6) |
| MAP SD | 8.7 (7.4-10.7) | 10.1 (8.5-12.9) |
| MAP CV | 9.8 (9.1-11.8) | 11.1 (8.8-13.5) |
| Mean PP | 49.3 (40.7-61.1) | 57.8 (47.7-66.0) |
| PP SD | 9.9 (8.6-13.0) | 12.2 (9.9-14.7) |
| PP CV | 20.0 (16.7-23.8) | 21.1 (18.2-26.5) |
| Percentage of SBP <140 mm Hg (per 10 percentage) | 93.3 (58.3-100.0) | 70.3 (43.1-91.3) |
| Percentage of DBP <70 mm Hg (per 10 percentage) | 35.6 (18.1-67.4) | 29.6 (13.3-62.3) |
| Percentage of MAP <90 mm Hg (per 10 percentage) | 54.2 (21.5-80.0) | 39.4 (14.5-62.0) |
